# The impact of social status inconsistency on cardiovascular risk factors, myocardial infarction and stroke in the EPIC-Heidelberg cohort

**DOI:** 10.1186/1471-2458-11-104

**Published:** 2011-02-16

**Authors:** Stefanie Braig, Richard Peter, Gabriele Nagel, Silke Hermann, Sabine Rohrmann, Jakob Linseisen

**Affiliations:** 1 Ulm University, Institute of Epidemiology and Medical Biometry, Germany; 2 Division of Cancer Epidemiology, German Cancer Research Centre, Heidelberg, Germany; 3 Institute of Epidemiology, Helmholtz Centre Munich, Neuherberg, Germany

## Abstract

**Background:**

Social inequalities in cardiovascular diseases are well documented. Yet, the relation of social status inconsistency (having different ranks in two or more status indicators like education, occupational position or income) and medical conditions of heart or vessels is not clear. Status inconsistency (SI) is assumed to be stressful, and the association of psychosocial distress and health is well known. Therefore, we aimed to analyze the relationship between cardiovascular diseases (CVD) and status inconsistency. Another target was to assess the influence of behaviour related risk factors on this association.

**Methods:**

8960 men and 6070 women, aged 45-65 years, from the EPIC-Heidelberg cohort (European Prospective Investigation into Cancer and Nutrition) were included. Socio-economic status was assessed by education/vocational training and occupational position at recruitment. During a median follow-up of 8.7 years, information on CVD was collected.

**Results:**

Compared to status consistent subjects, men who were in a higher occupational position than could be expected given their educational attainment had a nearly two-fold increased incidence of CVD (Odds Ratio (OR) = 1.8, 95% Confidence Interval (CI) = 1.5; 2.4, adjusted for age). Smoking behaviour and BMI differed significantly between those who had adequate occupational positions and those who did not. Yet, these lifestyle factors, as opposed to age, did not contribute to the observed differences in CVD. No association of cardiovascular diseases and status inconsistency was found for women or in cases where education exceeded occupational position.

**Conclusions:**

Status inconsistent men (occupational position > education) had a higher risk of cardiovascular diseases than status consistent men. However, harmful behaviour did not explain this relationship.

## Background

Cardiovascular diseases (CVD) are a major health problem in developed countries. According to the WHO, about 17 million people die of CVD every year, particularly of myocardial infarctions (MI) and strokes [1]. In addition to the established relationship between CVD and risk factors, a substantial number of studies report a strong inverse association of social status and mortality or morbidity of CVD [2-5].

Educational attainment, occupational position and income are established indicators of social status, indicating the (relative) rank an individual holds in society. Each of the above indicators has been shown to be inversely associated with CVD. Yet, it is not clear whether status inconsistency (SI), i.e. occupying discrepant positions in two or more of these ranking systems, affects health. One prominent example of such a mismatch is a university graduate working as a taxi driver. Status inconsistency is common in modern societies [6]. A low correlation between education, occupation, and income is regarded as a consequence of modernization, and increased welfare. A revolutionary improvement of living conditions, particularly of blue collar workers contributed to this development [7,8]. The transfer of status inheritance mechanisms from family to school system allows social mobility, one basic fundament of SI [9]. Additionally, high rates of unemployment and precarious working conditions may be causal for SI, since they force employees to accept inadequate occupational positions. Starting with Lenski (1954) [10], it is postulated that SI is associated with psychological distress, since individuals who made greater investments (higher education) do not obtain adequate rewards (income). This fact is seen as a violation of the theory of distributive justice [11]. From the viewpoint of role theory, SI is thought to be stressful as social interactions are disturbed. If, given the above mentioned example, one person interacts with his counterpart as if he was a taxi driver whereas the latter considers himself as a university graduate, the interaction would not succeed. SI can further be considered as relative deprivation [12,13]. In case other members belonging to the same social group are better off compared to oneself, and aspired goods are within reach, subjects may feel relatively deprived. Those who are status inconsistent in terms of being high rewarded though not adequately educated may feel guilty of being overrewarded. House & Harkins (1985) underlined the struggle necessary to maintain a high occupational status, and a feeling of being overloaded [14].

Psychosocial distress, on the other hand, is regarded as being causally related to CVD [15,16]. Nonetheless, studies found mixed evidence concerning the stressful effect of SI [14,17]. Furthermore, older empirical results have given little support to the hypothesis that status inconsistency is associated with medical conditions. This might be due to the design of these studies, the theoretical background, and the status indicators that were used to quantify SI among others (see [18,19] for an overview). Indeed, recent analyses showed an association between high education/low income and mental disorders [20], a significant decline in self rated health [21], and a significantly increased risk for mortality caused by coronary heart disease [22]. A current study indicated that the risk of ischemic heart disease is 3 times higher when education/training exceeds occupational position [23]. In contrast, articles focusing on status inconsistency and health related behaviour suggested mixed results [24,25].

To our knowledge, only little research has been conducted to examine the linkage between status inconsistency, health related behaviour and medical conditions. Thus, the aim of our study was (1) to investigate the association between social status inconsistency and CVD (MI, stroke) in the general population. Further (2), we wanted to explore which proportion of this association is explained by behavioural factors.

## Methods

### The EPIC Study

Data were gathered from the European Prospective Investigation into Cancer and Nutrition (EPIC) Study in Heidelberg, one of the largest cohort studies on the influence of diet and lifestyle factors on chronic diseases. Details of recruitment and follow-up procedures were described elsewhere [26,27]. Briefly, subjects (men aged 40-64 and women aged 35-64) were recruited during a period of four years (1994-1998). A random sample of individuals was drawn from general population registries. Information on lifestyle factors (alcohol consumption, smoking history, physical activity and diet), anthropometric data, information on medical history, and incident chronic diseases were collected at recruitment. Participation rate in Heidelberg was 38.3% compared to the invited number of subjects (n = 66 626).

Approximately every two years a follow-up questionnaire was mailed to the study participants, asking for diagnosed chronic conditions and the year of diagnosis. The participation rate in all follow-up rounds was more than 90% of all eligible study participants. The investigation was approved by the ethics committee at the University of Heidelberg.

For the present paper, we limited our study sample to subjects 40 to 65 years of age who held at least a half time employment at recruitment. Thus, the analytical cohort comprised 15980 subjects with a median follow up period of 8.7 years.

### Cardiovascular diseases

At recruitment, diagnoses of myocardial infarction (MI) and stroke were recorded by means of a self-administered questionnaire, asking whether a doctor ever had diagnosed MI or stroke. In the following years incident CVD events were identified by follow-up with questionnaire or mortality registers. Thereby, non-fatal events were ascertained by record linkage and reviews of medical records.

After the exclusion of subjects with prevalent events at baseline (69 cases of stroke, 183 of MI, 3 cases with MI and stroke), we identified the following number of events: 185 cases of stroke, 280 of MI, 12 cases with stroke and MI. We excluded further cases (13 cases of stroke, 62 of MI, 2 both events) where the status of events (incident or prevalent event) was unknown. For reasons of small numbers, we collapsed the two outcome variables under the headline cardiovascular diseases.

### Occupational attainment

Occupational position, recorded in twenty categories, was collapsed into 5 groups: 1 = un-skilled, semi-skilled manual or non-manual workers, 2 = skilled manual workers or non-manual workers with simple task, 3 = non-manual workers with middle task, middle level civil service, 4 = intermediates and 5 = professionals. Since the occupational status of farmers and family workers is ambiguous, we excluded subjects with these professions from the analyses.

### Educational attainment and vocational training

Education was measured according to the German school system. We completed the information by using information on vocational training. Similarly to occupational status, we divided education/vocational training into 5 classes, namely, 1 = < 10 years of education without vocational training, 2 = < 10 years with vocational training, 3 = > = 10 years without vocational training, 4 = > = 10 years with vocational training, 5 = university [28].

### Status inconsistency

SI was calculated according to Peter, Gässler, Geyer (2007) [23] by subtracting educational attainment/vocational training from occupational position, both categorized on the above-mentioned scales. Thus, SI indicated a difference in the position on two social ranks. In the following, we considered a difference of > = 2 points as SI. The distribution of SI is presented in table 1.

**Table 1:** Sociodemographic characteristics, cardiovascular disease morbidity, and distribution of CVD risk factors for the EPIC-Heidelberg cohort, 45-65 years of age, by sex.

	Men (N = 8960)	Women (N = 6070)
**Exposition**		
**Education, n (%)**		
< 10 years without vocational training	154 (1.7)	399 (6.7)
< 10 years with vocational training	3333 (37.3)	1838 (30.3)
> = 10 years without vocational training	360 (4.0)	383 (6.3)
> = 10 years with vocational training	1399 (15.7)	1629 (26.9)
University	3693 (41.3)	1813 (29.9)
Missing	21	8
**Occupational position, n (%)**		
Unskilled/semi-skilled worker, non-manuals	315 (3.6)	377 (6.3)
Skilled manuals or white collar workers with simple task	1300 (14.7)	887 (14.8)
White collar workers with middle task, middle level civil service	1521 (17.2)	2160 (26.1)
Intermediates, high level civil service	4906 (55.4)	2328 (38.9)
High qualified professional, Managerial, executive civil service	820 (9.3)	232 (3.9)
Missing	98	86
**Status inconsistency, n (%)**		
No status inconsistency	7000 (79.2)	4965 (83.1)
Education > Occupation	**505 (5.7)**	**535 (9.0)**
Education < Occupation	**1336 (15.1)**	**477 (8.0)**
Missing	119	93
**Confounder**		
**Age, n (%)**		
< 50 years	4318 (48.2)	3347 (55.1)
> = 50 years	4642 (51.8)	2723 (44.9)
Missing	0	0
**Diabetes, n (%)**		
Yes	196 (2.2)	58 (1.0)
No	8764 (97.8)	6012 (99.0)
Missing	0	0
**Smoking, n (%)**		
Never, former	6810 (76.0)	4587 (75.6)
Current	2150 (24.0)	1483 (24.4)
Missing	0	0
**BMI (kg/m^2^), Mean (Std)**	26.7 (3.6)	25.3 (4.6)
Missing	10	1
**Physical activity n (%)**		
Moderately active, active	2438 (28.1)	2600 (45.3)
Inactive, moderately inactive	6251 (71.9)	3139 (54.7)
Missing	271	331

### Covariates

Further covariates hypothesized to be potential confounders of the relationship between SI and CVD were introduced in multivariate analyses. Age was collapsed into two groups (1 = <50 years, 2 = > = 50 years). Smoking status was self-reported and classified into 0 = never/former smoker and 1 = current smoker. Physical activity was classified as 1 = inactive or moderately inactive vs. 0 = moderately active and active, based on occupational activity, cycling, and sports [29]. Elevated body mass index (BMI) was defined as 28.6 kg/m^2 ^or higher for men, and 27.5 kg/m^2 ^or higher for women respectively, which corresponded to the 75^th ^percentile of those who were not affected by CVD. We also used the 75^th ^percentile of the healthy population as cut-off point for dichotomising the alcohol consumption. Diabetes was self-reported and dichotomized into reported or not (1 = reported, 0 = not reported).

### Statistical analysis

Analyses were performed stratified by sex. Odds Ratios (OR) and 95% confidence intervals (95%-CI) are presented for the associations between SI and CVD. In order to quantify the impact of status inconsistency on health behaviour and BMI, we calculated ORs, considering behaviour related risk factors and BMI as outcome and status inconsistency as independent variable. SAS statistical software 9.13 (SAS institute Inc, Cary, NC) was used for all calculations.

## Results

Baseline data on 6070 Women and 8960 Men are displayed in Table 1.

Subjects analyzed were well educated and had elevated occupational positions.

Marked gender differences were found in the distribution of status inconsistency. SI in terms of higher occupational position compared to education was more prevalent for men (15.1%) than for women (8.0%). On the other hand, a combination of high education and low occupation was predominant among women (9.0% vs. 5.7% in men). Younger subjects were more likely to be status inconsistent in terms of being higher qualified compared to their occupation (data not shown in the table). With regard to the other groups, men with low education occupying higher occupational positions showed the highest incidence of CVD (7.2%, p (Chi square) < 0.001) (see table 2).

**Table 2:** Cardiovascular disease morbidity for the EPIC-Heidelberg cohort, 45-65 years of age by sex.

	Men (N = 8960)	Women (N = 6070)
**MI, n (%)**	241 (2.7)	39 (0.6)
**Stroke, n (%)**	137 (1.5)	48 (0.8)
**Both, MI and Stroke, n (%)**	10 (0.1)	2 (-)
**Total CVD, n (%)**	388 (4.3)	89 (1.5)
		
**CVD by status inconsistency**		
**CVD by status inconsistency, n/n exposed (%)**		
No status inconsistency	263/7000 (3.8)	77/4965 (1.6)
Education > Occupation	22/505 (4.4)	4/535 (0.8)
Education < Occupation	96/1336 (7.2)	7/477 (1.5)
Missing on SI	7	1

Contrary to status inconsistency, the impact of "traditionally" used indicators of social status (occupational position, educational attainment) on CVD was only limited (see table 3). Compared to subjects who graduated from university, those with lower education had a higher risk of CVD, albeit only few associations reached significance. No significant association between occupational status and CVD was found.

**Table 3:** Association between CVD, occupational status and educational attainment (OR, 95% CI) in the EPIC-Heidelberg cohort, 45-65 years of age, among men and women adjusted for age.

	Men	Women
	OR (95% CI)	OR (95% CI)
**Occupational status**		
Unskilled/semi-skilled workers, non-manuals	1.5 (0.8; 2.7)	1.2 (0.4, 3.9)
Skilled manuals or white collar workers with simple task	1.2 (0.8; 1.9)	0.8 (0.3, 2.4)
White collar workers with middle task, middle level civil service,	1.1 (0.7, 1.7)	0.9 (0.3; 2.5)
Intermediates, high level civil service	0.9 (0.7, 1.4)	0.7 (0.2; 1.9)
High qualified professional, Managerial, executive civil service	1.0	1.0
**Education**		
< 10 years without vocational training	1.9 (0.9; 3.7)	**2.3 (1.0; 5.1)**
< 10 years with vocational training	**1.8 (1.5; 2.3)**	1.8 (1.0; 3.3)
> = 10 years without vocational training	1.1 (0.6; 2.1)	1.9 (0.7; 4.9)
> = 10 years with vocational training	1.1 (0.8; 1.6)	1.6 (0.9; 3.1)
University	1.0	1.0

Table 4 reflects behavioural factors and BMI supposed to be associated with social status inconsistency. Compared to men without SI, status inconsistent men were more likely to smoke (education > occupation: OR = 1.5, (95%CI = 1.2; 1.8), education < occupation: OR = 1.2, (95%CI = 1.0; 1.4)). For women, only high occupational position combined with low educational attainment was linked to a higher risk of smoking. Overweight was associated with status groups characterized by low education in combination with high occupation. In men, SI (education < occupation) was further associated with a smaller risk of being physically inactive (OR = 0.8, 95%CI = 0.7; 0.9).

**Table 4:** Association between potential confounder and status inconsistency (OR, 95% CI) in the EPIC-Heidelberg cohort, 45-65 years of age, among men and women adjusted for age.

	Men	Women
	OR (95% CI)	OR (95% CI)
**Current smoker**		
No status inconsistency	1.0	1.0
Education > Occupation	**1.5 (1.2; 1.8)**	0.9 (0.7; 1.1)
Education < Occupation	1.2 (1.0; 1.4)	**1.4 (1.1; 1.7)**
**Inactive/moderately inactive**		
No status inconsistency	1.0	1.0
Education > Occupation	0.9 (0.7; 1.1)	0.9 (0.8; 1.1)
Education < Occupation	**0.8 (0.7; 0.9)**	0.8 (0.7; 1.0)
**BMI > 75^th ^percentile**		
No status inconsistency	1.0	1.0
Education > Occupation	0.8 (0.7; 1.0)	0.9 (0.7; 1.1)
Education < Occupation	**1.6 (1.4; 1.8)**	**1.6 (1.3; 2.0)**
**Alcohol consumption > 75^th ^percentile**		
No status inconsistency	1.0	1.0
Education > Occupation	0.9 (0.8; 1.2)	1.0 (0.8; 1.2)
Education < Occupation	1.0 (0.9; 1.1)	0.9 (0.7; 1.2)
**Diabetes**		
No status inconsistency	1.0	1.0
Education > Occupation	0.9 (0.4; 1.8)	0.8 (0.3; 2.3)
Education < Occupation	1.2 (0.8; 1.7)	1.5 (0.7; 3.3)

Table 5 displays the bi- and multivariate associations between CVD and status inconsistency adjusted for CVD risk factors. When occupational rank was higher compared to the educational level, the risk of CVD was 1.8 times higher than for those men without SI (95%CI = 1.5; 2.4, adjusted for age). There were no significant results, neither for women nor for the second form of status inconsistency (education > occupation).

**Table 5:** Association between CVD and status inconsistency, crude and adjusted for risk factors (OR, 95% CI) in the EPIC-Heidelberg cohort, 45-65 years of age.

	Men	Women
	OR (95% CI)	OR (95% CI)
**Crude**		
No status inconsistency	1.0	1.0
Education > Occupation	1.2 (0.7; 1.8)	0.5 (0.2; 1.3)
Education < Occupation	**2.0 (1.6; 2.5)**	0.9 (0.4; 2.1)
**Adjusted for Age (cat.)**		
No status inconsistency	1.0	1.0
Education > Occupation	1.3 (0.8; 2.0)	0.5 (0.2; 1.3)
Education < Occupation	**1.8 (1.5; 2.4)**	0.9 (0.4; 1.9)
**Adjusted for Age and Smoking**		
No status inconsistency	1.0	1.0
Education > Occupation	1.2 (0.8; 1.9)	0.5 (0.2; 1.3)
Education < Occupation	**1.8 (1.5; 2.4)**	0.9 (0.4; 1.9)
**Adjusted for Age and physical** **activity**		
No status inconsistency	1.0	1.0
Education > Occupation	1.3 (0.8; 2.0)	0.4 (0.1; 1.2)
Education < Occupation	**1.9 (1.5; 2.4)**	0.8 (0.3; 1.8)
**Adjusted for Age and BMI (> = 75^th ^percentile)**		
No status inconsistency	1.0	1.0
Education > Occupation	1.3 (0.8; 2.1)	0.4 (0.2; 1.2)
Education < Occupation	**1.8 (1.4; 2.3)**	0.8 (0.3; 1.8)
**Adjusted for Age and Alcohol (> = 75^th ^percentile)**		
No status inconsistency	1.0	1. 0
Education > Occupation	1.3 (0.8; 2.0)	0.5 (0.2; 1.3)
Education < Occupation	**1.8 (1.5; 2.4)**	0.9 (0.4; 1.9)
**Adjusted for Age and Diabetes**		
No status inconsistency	1.0	1.0
Education > Occupation	1.3 (0.8; 2.1)	0.5 (0.2; 1.4)
Education < Occupation	**1.8 (1.4; 2.3)**	0.8 (0.4; 1.8)
**Adjusted for all covariates** **simultaneously**		
No status inconsistency	1.0	1.0
Education > Occupation	1.2 (0.8; 2.0)	0.4 (0.1; 1.3)
Education < Occupation	**1.8 (1.4; 2.3)**	0.7 (0.3; 1.6)

When adjusting for behavioural risk factors, the association between status inconsistency and CVD was not attenuated. The Nagelkerke *R^2 ^*= .057 suggests that the relationship between the CVD and the predictors included in the model is only small.

We repeated our analysis including TIA (transitory ischemic attack) into the composite outcome variable. Results did not differ substantially from those presented here.

## Discussion

In this study, low education in combination with high occupation was related to CVD in men, but not in women. Men with low educational status working in a higher occupational position had a nearly two-fold increased incidence of CVD than men without SI. Yet, we did not find any effect of high education combined with low occupation as hypothesized. Smoking behaviour, physical activity and BMI differed between status inconsistent individuals and the reference group. However, the effect of SI on cardiovascular health was not explained by health behaviour. The fit of respective models was limited.

The EPIC-Heidelberg sample was well educated and held high occupational positions at baseline which is characteristic of the population in Heidelberg. Heidelberg, located in the south west of Germany, is Germany's oldest university town with the country's highest percentage of graduates (more than 50%), a higher-than-average income, and a comparatively young population [26,30]. This may affect the incidence of CVD and the prevalence of status inconsistency in our study.

Concerning the outcome we found a restricted number of events, even though similar to the rate in Germany, given a mean follow-up period of nearly 9 years [31-33]. This small number was traced back on relatively low age of the study population.

Our results show 15% of men and 8% of women to be status inconsistent, with low education but relatively high occupational position. These rates are approximately in line with Groot, Maassen van den Brink 2000 [6] but lower as found by Peter et al. 2007 [23]. However, our study population differed substantially from that in the latter study with regard to education and occupational position.

We assume that the restricted number of SI and a small incidence of CVD in women could be one reason why we did not find an association of CVD and SI in female subjects.

The small number of events may also affect the associations between "traditionally examined" social indicators and CVD. Though the estimates of the risks showed a social gradient in the incidence of CVD, they did not reach statistical significance.

Although psychosocial distress is not measured in this study, several other investigations showed that SI is associated with increased psychosocial distress because of relative deprivation or distributive injustice (in case education exceeds occupational position). Psychological distress elicited by the other type of SI (education < occupational position) may be induced by work overload. However, to our knowledge the mediating effect of psychosocial factors in the association between SI and health has not been investigated so far. The mediating effect of *occupational distress *is currently studied by our group.

Hart et al. 1998 [34] found fewer smokers and wine drinkers among status inconsistent individuals (education < occupational position). Similar results were observed in the GAZEL study [25]. Additionally, the latter study indicated that status inconsistent men (education < occupational position) had a lower risk of being overweight than those without SI.

We found a higher risk for smoking and of being overweight in status inconsistent men. Although these results are not in line with the above mentioned studies, one could interpret these findings in the light of psychological distress. Episodes of higher stress levels are related to smoking [35], and weight gain as well as weight loss [36,37].

Concerning the relationship between status inconsistency and cardiovascular diseases, older studies showed conflicting results (see Vernon, Buffler 1988 [19]). Our findings partly supported one study conducted more recently [23], albeit we could not find the harmful effect of high education combined with low occupation, or the effect in the female study population.

Since our data come from a German population, the question has to be addressed, if the results can be extrapolated to countries outside of Germany. SI is regarded as a consequence of modernization (comprising individualisation and higher importance of meritocratic than heritage principles), thus we hypothesize that similar results can hold true for all modernized countries. Authors from the Czech Republic [38] underlined that SI is more prevalent in post-communistic countries, a hypothesis partly approved by Kohler 2005 [8] who further showed high rates of SI for Turkey. The stressful effect of SI is probably not limited to Western countries. The association between SI and health was also proven by Gal et al. 2008 in Israel [20].

EPIC-Heidelberg, one of the largest cohort studies of chronic diseases, is of particular interest because it provides longitudinal data from the general population. By excluding subjects with prevalent CVD before recruitment, reversed causality - career mobility caused by CVD morbidity - is excluded. Furthermore, to our knowledge, there is no other study analyzing the association of status inconsistency and health outcomes that are ascertained by reviews of medical records or mortality registers.

Some potential limitations of our study should be considered: First, due to a small number of events, we had to collapse two outcome variables, stroke and MI. Second, since data on income was not available in EPIC-Heidelberg we did not analyze further forms of SI arising, e.g. income exceeding occupational position/educational attainment or vice versa.

Our models only explained a small partition of the overall variability. Yet, the model fit is better compared to models using education/vocational training and occupational position as separate independent variables. Thus, we conclude that information on status inconsistency may help to further improve knowledge about social inequality in health.

There is a broad discussion concerning the correct model specification to quantify the effects of status inconsistency. Simpson 1985 [39] and Zhang 2008 [40] suggested to include both, the status variables and (multiplicative) interaction terms in one model. However, when we added the single status variables to our model, the effects of SI were attenuated but were still significant. Although we hypothesized an increased CVD risk among highly educated persons in low social positions we could not find such an association. We assume that there could be a selection effect, which affects the level of non-response in groups of low occupational position [41].

## Conclusions

Status inconsistent men had a higher risk of CVD than occupationally stable individuals, whereas we only found inconsistent associations of single indicators of social status with cardiovascular diseases. Health related risk factors like smoking and BMI were associated with status inconsistency. However, harmful behaviour did not explain the relationship between status inconsistency and CVD.

## Competing interests

The authors declare that they have no competing interests.

## Authors' contributions

GN and RP initiated and designed the project. JL is principal investigator of the EPIC-Heidelberg cohort study and contributed to the study design. SR and SH were involved in data collection and contributed to the variable selection. All authors provided specialist knowledge in the advancement of the analyses and provided input in re-drafting the manuscript. SB conducted the statistical analyses and wrote the manuscript, which was seen and approved by all authors.

## Funding

The EPIC-Heidelberg study was funded by "Europe Against Cancer" Program of the Euro-pean Commission (SANCO); German Cancer Aid; German Cancer Research Center; German Federal Ministry of Education and Research, and Kurt-Eberhard-Bode-Stiftung.

## Pre-publication history

The pre-publication history for this paper can be accessed here:

http://www.biomedcentral.com/1471-2458/11/104/prepub
